# The significance of serum uric acid level in humans with acute paraquat poisoning

**DOI:** 10.1038/srep09168

**Published:** 2015-03-16

**Authors:** JuanWen Zhang, Ying Zhao, YunJuan Bai, GuoCai Lv, JianPing Wu, Yu Chen

**Affiliations:** 1 Department of Laboratory Medicine, The First Affiliated Hospital, College of Medicine, Zhejiang University, 79 Qingchun Road, Hangzhou 310003, China

## Abstract

Hyperuricemia is a strong and independent predictor of all-cause mortality in cardiovascular disease and has been found to play a role in diseases exacerbated by oxidative stress and inflammation. This study aimed to evaluate whether serum uric acid (UA) level is an indicator of outcome in patients with acute paraquat poisoning. A total of 205 subjects who had attempted suicide by oral ingestion of paraquat were admitted to the emergency room between January 2009 and June 2014. Initial serum UA level and other laboratory parameters were measured. A total of 66 patients died during the 30 days after admission, corresponding to a 32.2% cumulative incidence of mortality. UA levels were higher in non-survivors than survivors (*P* < 0.001) and 30-day mortality increased with increasing baseline serum UA level (*P* < 0.001). In a prediction analysis for 30-day mortality, the serum UA level had a cut-off concentration of 284 µmol/L in female patients and 352 µmol/L in male patients. Multivariate Cox proportional hazards regression analyses showed that white blood cell counts and UA were independent prognostic factors. In conclusion, we showed that serum UA may be an independent predictor of 30-day mortality in patients with paraquat poisoning.

Paraquat (PQ) is a bipyridyl, rapidly acting, nonselective herbicide widely used in the developing world^1^,^2^. It is a highly toxic compound to human beings, with no known antidote^3^,^4^. Intentional or accidental acute PQ poisoning is unfortunately common and many fatal cases have been reported in China^5^,^6^. Poisoning cases are most often a result of suicide attempts via oral self-administration^7^.

PQ is largely secreted unchanged in urine within the first 24 hours of ingestion. To date, the most widely accepted mechanism underlying PQ intoxication is oxidative stress. Biotransformation of PQ in cells results in the generation of superoxide anions and subsequently other free radicals, resulting in cellular injury such as lipid peroxidation and mitochondrial dysfunction, triggering an inflammatory response^8^,^9^,^10^. Uric acid (UA) is the major end product of purine metabolism and is formed from hypoxanthine and xanthine by the rate-limiting enzymatic action of xanthine oxidoreductase (XO)^11^,^12^. It has been reported that PQ treatment increases XO activity and stimulates hypoxanthine-dependent superoxide production in the cytosol of rat lungs^13^,^14^. Our previous study indicated increased XO activity accompanied by lipid peroxidation and reduced total antioxidant capacity in subjects with acute PQ poisoning^15^.

Paraquat poisoning is characterized by multiple organ function failure, mainly involving the lung, kidney, heart, liver, and nervous system^16^. Yu et al. observed that treatment with UA could protect neurons against excitotoxic and metabolic insults involving suppression of oxyradical accumulation, stabilization of calcium homeostasis, and preservation of mitochondrial function^17^. Conversely, Sakai and colleagues reported that the use of allopurinol as a drug to block the production of UA can alleviate intracellular free radical production and reduce PQ cytotoxicity in cultured bovine pulmonary artery endothelial cells^18^. A recent study also found that basal levels of UA in mice do not appreciably protect against oxidative damage and neurotoxicity in the PQ model of Parkinson's disease^19^. To date, any association between UA and PQ exposure remains uncertain in the literature. Our hypothesis is that elevated UA is a valuable prognostic factor for adverse outcomes after PQ poisoning. Because of a lack of specific antidotes, the overall mortality from acute PQ poisoning is substantially high^20^. This raises the need to develop a valuable predictor for prognosis to guide future therapeutic intervention. Further clarification of serum UA levels in patients with PQ poisoning may thus have significant clinical implications by setting a framework for modulating serum UA levels.

## Methods

### Subjects

This study was a retrospective observational cohort study of patients presenting to the emergency room (ER) of The First Affiliated Hospital, College of Medicine, Zhejiang University between January 2009 and June 2014. We enrolled 221 patients with an oral intake of paraquat from 2 to 30 mL. Patients who met the following criteria were excluded: those with a history of gout (n = 8), diabetes mellitus (n = 3), hypertension (n = 2), renal failure (n = 2), and malignancy (n = 1). The remaining 205 patients [median age: 33.0 years (range: 14–82 years); female patients: 110; male patients: 95] were included in the present study. Because UA concentrations differ significantly by gender, patients were categorized into gender-specific tertiles based on their UA level: tertile 1, UA < 329 µmol/L for men and UA < 237 µmol/L for women; tertile 2, 329–431 µmol/L for men and UA 237–334 µmol/L for women; tertile 3, UA > 431 µmol/L for men and UA > 334 µmol/L for women. Informed consent was obtained from all the subjects.

### Sample collection and biochemical analyses

A peripheral venous blood sample (6 mL) was collected from each patient within the first 24 hours after admission to the ER. Blood samples were used to analyze the hematological index and biochemical values. Laboratory parameters measured included: white blood cells (WBC), platelets, hemoglobin, red blood cell distribution width (RDW), neutrophil-lymphocyte ratio (NLR), prothrombin time, total protein, albumin, alanine aminotransferase (ALT), aspartate aminotransferase (AST), lactate dehydrogenase (LDH), creatine kinase (CK), creatinine, potassium, pH, PaCO_2_, and PaO_2_. All biochemical analyses were conducted using a Hitachi 7600 Clinical Analyzer (Hitachi, Tokyo, Japan), Sysmex CA-7000 System (Sysmex, Kobe, Japan), and Sysmex XE-2100 Automated Analyzer (Sysmex) using standard methods.

### Data and statistical analysis

Statistical analyses were performed using SPSS, version 16 (SPSS, Chicago, IL, USA). Data are presented as the mean ± standard deviation when data were found to be normally distributed or as the median if the distribution was skewed. The differences among multiple groups or between two groups were assessed using a one-way analysis of variance (ANOVA) and the Kruskal–Wallis H test or Mann–Whitney U test, if appropriate. Differences by gender and in 30-day mortality among the groups were compared using a Chi-squared test. The area under the ROC curve was used to discriminate UA levels with respect to 30-day mortality. Univariate and multivariate Cox regression analyses to determine predictors of 30-day mortality were presented as hazard ratios with a 95% confidence interval. Variables that showed a *P* value < 0.05 in the univariate analysis were included in the multivariate analysis. All statistical tests were two-tailed. *P* < 0.05 was considered significantly different.

### Ethics statement

This study was approved by the ethics committee of The First Affiliated Hospital, College of Medicine, Zhejiang University and was conducted in accordance with the Declaration of Helsinki.

## Results

### Patient characteristics

We divided patients with PQ poisoning into three groups [Tertile 1 (lowest), Tertile 2, and Tertile 3 (highest)] according to the tertile of their serum UA levels. As shown in Table 1, we found that time from PQ ingestion to ER admission was significantly different between the lower two tertiles and the highest tertile. Across increasing serum UA tertiles, WBC, NLR, ALT, AST, LDH, CK, and creatinine levels were gradually increased, while potassium, arterial pH, and PaCO_2_ gradually decreased.

### Association between serum UA level and biochemical variables

Serum UA levels were significantly and positively correlated with inflammatory indexes [WBC (r = 0.196, *P* < 0.05), LDH (r = 0.178, *P* < 0.05), and NLR (r = 0.173, *P* < 0.05)] and markers of multi-organ damage [ALT (r = 0.171, *P* < 0.05), AST (r = 0.192, *P* < 0.05), CK (r = 0.186, *P* < 0.05), and creatinine (r = 0.398, *P* < 0.05)]. Serum UA levels were also negatively correlated with parameters of arterial blood gases [pH (r = −0.161, *P* < 0.05) and PaCO_2_ (r = −0.171, *P* < 0.05)] and potassium (r = −0.182, *P* < 0.05).

### Comparison of the serum UA level between the survival group and non-survival group

**Figure 1:**
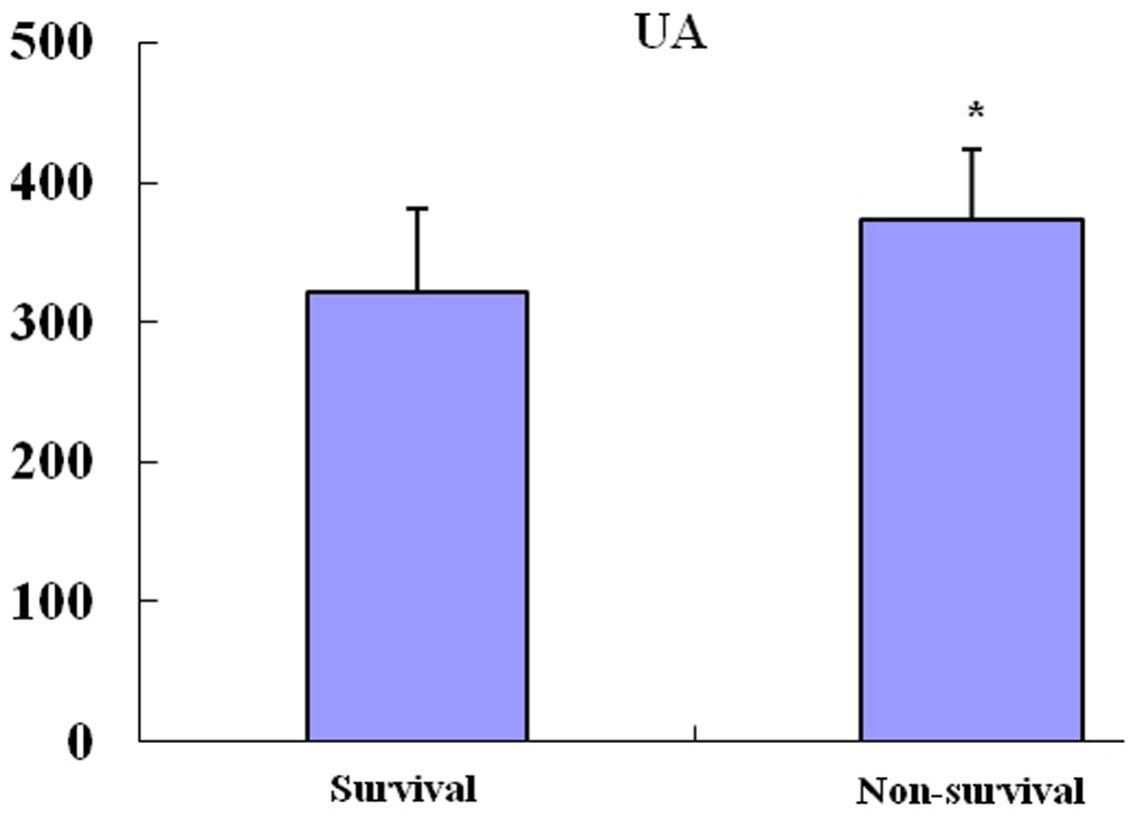
Comparison of serum UA level between the survival group and non-survival group. Data are mean ± SD. *P < 0.05 compared with the survival group.

As shown in Figure 1, the serum UA level in the non-survival group was significantly higher than in those who survived (373.3 ± 51.2 µmol/L *vs* 321.6 ± 60.1 µmol/L, *P* < 0.001).

### Association of UA level with 30-day mortality rate

Sixty-six patients died during the first 30 days after admission to the ER, corresponding to a 32.2% cumulative incidence of mortality. To get a deeper understanding of the relationship between UA level and PQ poisoning, the cumulative 30-day mortality of patients was calculated by dividing the number of fatalities by the number of subjects in each UA tertile (Table 2). The 30-day mortality rate tended to increase as the UA level increased. Compared with only 17.1% in tertile 1, the 30-day mortality rates for the subjects in tertile 2 and tertile 3 were 32.4% and 47.8%, respectively.

### Optimal UA cut-off value for predicting 30-day mortality

**Figure 2:**
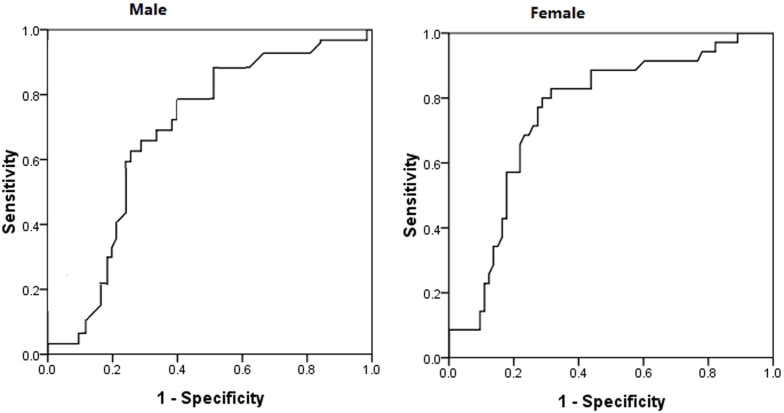
Cut-off point for UA for predicting 30-day mortality from paraquat poisoning.

The cut-off point for UA to predict 30-day mortality in female patients was 284 µmol/L and the area under the receiver operating characteristic (ROC) curve was 0.752 (95% confidence interval, 0.655–0.849, *P* < 0.001) (Figure 2). When the UA was >284 µmol/L in female patients, the sensitivity was 82.9%, and the specificity was 68.5%. The cut-off point for UA in male patients was 352 µmol/L and the area under the ROC curve was 0.732 (95% confidence interval, 0.637–0.829, *P* < 0.001). The sensitivity was 79.2% and the specificity was 60.5%.

### Risk factor analysis for 30-day mortality

As shown in Table 3, univariate analysis showed a significant association of WBC, creatinine, LDH, CK, and UA with 30-day mortality. In the multivariate Cox proportional hazards regression analyses, WBC and UA were independent prognostic factors.

## Discussion

Various studies have shown that PQ primarily exerts its toxic effects through the redox cycle, which produces oxygen free radicals, leading to oxidative damage and eventual cell death^21^,^22^,^23^. Many studies have sought to evaluate outcome indicators of PQ intoxication, but there is still no consensus on a practical indicator. To date, no prognostic models have been prospectively validated because of issues in their development such as small sample size, differences in the degree of severity, and complicated exclusion criteria^24^. The urine dithionite test is a simple index for clinical diagnosis and prediction of prognosis of PQ poisoning; however, false results limit its usefulness^25^. Our study is one of the few performed to date addressing serum UA levels and laboratory parameters in a clinical context with relatively large sample size. Our results confirmed our hypothesis: 30-day mortality increased with progressively higher baseline serum UA level and increased UA level was found to be an independent prognostic factor in patients with acute PQ poisoning. Recently, there has been growing interest in this parameter because increased UA is a strong independent biomarker of adverse outcomes in many diseases and conditions linked to increased oxidative stress and inflammation^26^,^27^,^28^. UA is formed via the purine degradation pathway by the action of XO and is excreted by the kidney into the urine^29^,^30^. Serum UA levels are rigorously controlled by a balance between UA synthesis and excretion. One of the plausible explanations for an increased UA level in human PQ poisoning is upregulation of XO activity. Studies have confirmed that PQ treatment significantly induces XO activity and increases hypoxanthine-dependent superoxide production^13^,^14^,^18^, which would then result in increased UA as it is the other main product of XO activity. Similarly, we previously reported significantly higher serum XO activity in a PQ poisoning group vs healthy controls^15^. In addition to the increased generation of UA, another possible mechanism for a rise in serum UA is reduced excretion. The kidney is considered the main excretory organ for PQ in humans, eliminating it in the form of urine^31^. Robust epidemiological data have shown PQ-induced kidney injury such as acute tubular necrosis in the proximal tubule, interstitial inflammation, and impaired glomerular filtration rate^32^,^33^. Renal dysfunction leads, in turn, to decreased serum UA clearance.

These results provide novel evidence for a strong association between UA and the mechanism of PQ toxicity. Several mechanisms could explain the significant relationships between serum UA and mortality as a result of PQ poisoning. The association between hyperuricemia and increased risk of metabolic syndrome and cardiovascular disease has been evaluated, and hyperuricemia has been shown to be a strong and independent predictor of all-cause mortality in community-based studies^34^,^35^. Over the past few years, epidemiological studies have repeatedly demonstrated that UA acts as a strong oxidant and an elevated serum UA level may stimulate oxidative stress and endothelial dysfunction in several pathological states^36^,^37^. UA also triggers an inflammatory response by stimulating the production of proinflammatory cytokines^38^. Increased systemic oxidative stress and inflammation play a crucial role in the development of PQ-induced injury both in animal experiments and clinical studies^39^,^40^,^41^. The correlation between UA and inflammatory indices and markers of organ damage in the present study also support that UA may be a significant risk factor for the development of PQ poisoning. Further investigation regarding the association between UA and PQ poisoning will expand our understanding of this toxicity.

This study has some limitations. First, we can only propose a role for UA in the etiology of PQ poisoning based on a snapshot of the circulating UA state. This observational study was unable to definitively comment on causality or the temporal association between high serum UA and PQ poisoning. The second limitation is that blood PQ levels were not assessed and PQ poisoning cases were included on the basis of a history of oral PQ ingestion. Although, the prognostic value of plasma PQ has been previously documented in subjects with acute PQ poisoning^42^,^43^. Unfortunately, this assay is not commonly available in the ER owing to limited medical facilities^44^. In addition, traditional methods for detecting PQ levels are time consuming and most ER physicians do not rely on the results of plasma PQ measurements for emergency management decisions^45^.

In summary, our results demonstrated that measurement of serum UA may be a simple and practical index for assessing the outcome of PQ poisoning. Based on this finding, interventions that aim to decrease UA levels may have beneficial effects in treating patients with acute PQ intoxication. Nevertheless, further studies verifying the precise role of UA are needed to eventually guide development of clinical intervention strategies for PQ poisoning.

## Author Contributions

Y.C. designed the experiments. Y.J.B., G.C.L. and J.P.W. performed the experiments. J.W.Z. and Y.Z. wrote the majority of the manuscript text. All authors reviewed the final manuscript.

## Figures and Tables

**Table 1:** Baseline laboratory characteristics according to UA tertile

Variable	Tertile 1 (n = 70)	Tertile 2 (n = 68)	Tertile 3 (n = 67)	*P* value
Age (yr)	31.0(24–68)	34.0(14–71)	32.5(15–82)	0.276
Gender (male/female, n)	32/38	32/36	31/36	0.987
Time from ingestion to ER (hr)	6.6 ± 4.9	6.9 ± 7.1	11.6 ± 8.1	0.026
WBC (10^9^/L)	11.5(4.0–28.6)	13.6(4.1–42.3)	14.6(4.8–48.6)	< 0.001
Platelet(10^9^/L)	193.6 ± 74.1	211.6 ± 80.1	206.6 ± 74.1	0.301
Hemoglobin (g/L)	136.6 ± 18.1	137.1 ± 19.2	136.8 ± 20.1	0.872
RDW (%)	12.8(11.4–17.1)	12.6(11.4–20.1)	12.7(11.2–16.2)	0.346
NLR	9.2(1.1–42.1)	9.8 (1.3–53.2)	13.1 (1.6–62.2)	0.031
PT(s)	11.7(9.9–22.1)	11.6(9.5–26.1)	11.8(9.4–29.7)	0.289
Total protein (g/L)	69.6 ± 7.1	69.0 ± 6.5	69.4 ± 7.5	0.802
Albumin(g/L)	44.6 ± 5.1	44.1 ± 4.3	43.2 ± 4.7	0.813
ALT (U/L)	16(5–342)	22(6–405)	23(10–601)	0.030
AST (U/L)	22(11–140)	24(13–463)	28(14–623)	< 0.001
LDH (U/L)	201(112–430)	233(113–650)	238(118–826)	0.004
CK(U/L)	122(42–320)	132(52–733)	149(59–930)	0.027
Creatinine (umol/L)	58(24–169)	67(32–201)	118(42–279)	< 0.001
Potassium (mmol/L)	3.79 ± 0.48	3.52 ± 0.52	3.39 ± 0.57	0.029
PH	7.43(7.25–7.57)	7.41(7.19–7.51)	7.40(7.11–7.50)	0.005
PaCO_2_ (mmHg)	31.9 ± 6.7	30.7 ± 7.7	28.1 ± 7.2	0.014
PaO_2_ (mmHg)	101.6 ± 26.7	102.6 ± 31.7	98.6 ± 36.7	0.532

Abbreviations: WBC, white blood cell; RDW, red blood cell distribution width, NLR, neutrophil-lymphocyte ratio; PT, prothrombin time; ALT, alanine aminotransferase; AST, aspartate aminotransferase; LDH, lactate dehydrogenase; CK, creatine kinase.

**Table 2:** 30-day mortality according to serum UA tertile

UA Tertile	Total	Fatalities	30-day mortality (%)	X^2^	*P* value
Tertile 1	70	12	17.1		
Tertile 2	68	22	32.4	4.298	0.038
Tertile 3	67	32	47.8	14.720	<0.001

**Table 3:** Cox regression analysis of risk factors for 30-day mortality in patients with PQ poisoning

	Univariate		Multivariate	
	HR(95%CI)	P value	HR(95%CI)	P value
WBC	1.046(1.023–1.071)	<0.001	1.069(1.025–1.116)	0.002
Creatinine	1.002(1.001–1.004)	0.006	1.001(0.998–1.004)	0.619
LDH	1.003(1.002–1.004)	<0.001	0.999(0.996–1.002)	0.465
CK	1.000(1.000–1.001)	0.002	1.001(0.999–1.001)	0.097
UA	1.003(1.001–1.004)	0.002	1.001(1.001–1.003)	0.045
